# Quantum dissipative effects on non-equilibrium transport through a single-molecular transistor: The Anderson-Holstein-Caldeira-Leggett model

**DOI:** 10.1038/srep18511

**Published:** 2016-01-06

**Authors:** Ch. Narasimha Raju, Ashok Chatterjee

**Affiliations:** 1 School of Physics, University of Hyderabad, Hyderabad-500046, India

## Abstract

The Anderson-Holstein model with Caldeira-Leggett coupling with environment is considered to describe the damping effect in a single molecular transistor (SMT) which comprises a molecular quantum dot (with electron-phonon interaction) mounted on a substrate (environment) and coupled to metallic electrodes. The electron-phonon interaction is first eliminated using the Lang-Firsov transformation and the spectral density function, charge current and differential conductance are then calculated using the non-equilibrium Keldysh Green function technique. The effects of damping rate, and electron-electron and electron-phonon interactions on the transport properties of SMT are studied at zero temperature.

The subject of transport in single molecular transistors has attracted considerable attention in recent years primarily for their potential applications in nano-technology. A single molecular transistor (SMT) is an electronic device in which a central molecule or quantum dot (QD) connected by metal leads (source and drain), plays an important role in transport. The central molecule or the QD is characterized by its discrete energy levels with coulomb interaction. These SMTs can be used as single-electron transistors^1^ by controlling the charge transport using Coulomb blocked effect. The charging effects like Coulomb blockade and Kondo effect due to electron-electron (*el-el*) interaction in such systems is well understood^2^,^3^,^4^,^5^,^6^,^7^,^8^,^9^. Of late, the phononic effects on molecular devices have been studied by many research groups. Each electron-transfer from the lead to the molecule creates a distortion in the molecule. Quanta of this distortion, called phonons, interact with a local electron of the molecule through the electron-phonon (*el-ph*) interaction giving rise to what is known as the polaronic effect. Particularly in organic conjugated molecules and quantum dots, quasiparticles that take part in transport mechanism are polarons^10^,^11^,^12^. The transport properties of such systems are actually affected by both *el-el* and *el-ph* interactions. Much effort, therefore, has gone into understanding the quantum transport in SMT theoretically incorporating both *el-ph* and *el-el* interactions in different regimes. Many experimental and theoretical groups^13^,^14^,^15^,^16^ have demonstrated that *el-ph* interaction is the cause for the existence of the vibrational side bands. In Ref.^16^ Braig *et al.* have studied the effect of dissipative surroundings around the molecule using Rate equation approach in weak coupling limit. They have suggested that the effect of dissipative surroundings around the molecule is to provide an additional broadening of the vibrational side bands. Other theoretical methods applied in this field include the Kinetic equation method^17^,^18^, the rate equation approach^19^, non-equilibrium Green’s function approaches^20^,^21^,^22^ and numerical renormalization group method^23^,^24^. In the present work we study the damping effect in SMT device using the Keldysh mechanism. Our study is valid for the entire range of the coupling constant. We restrict ourselves only to zero temperature.

The paper is organized as follows: In the section immediately following, we introduce the model and the relevant Hamiltonian for the problem. To be more specific, we consider the Anderson-Holstein (AH) model with the Caldeira-Leggett term. In the next section i.e., in “Polaron Transformation” section, we apply the celebrated Lang-Firsov canonical transformation to eliminate the *el-ph* interaction in the first order. In the “Tunneling Current” section we determine the tunneling current, spectral function and the differential conductance using the Keldysh formalism. In the “Results and Discussion” section we discuss the numerical results and finally present our concluding remarks in the “Conclusion” section.

## The Model

**Figure 1:**
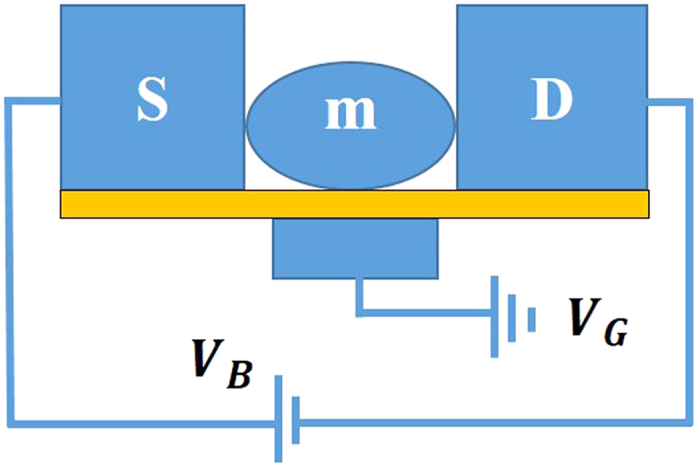
Schematic diagram of the SMT system considered in this paper. In the above diagram S, D and m refers source, drain and molecule respectively.

Figure 1 shows the schematic description of an SMT system. A typical SMT device consists of a single-level molecule or a QD coupled to two metal leads. The molecule is assumed to have a single vibrational mode interacting with its charge by *el-ph* interaction. The system is embedded on an insulating substrate (yellow colour part in Fig. 1) that can be approximated as a bath of independent harmonic oscillators in the spirit of the Caldeira-Leggett model. The substrate can cause a damping effect that can be described by a linear coupling term between the local phonon field of the molecule and a set of independent harmonic oscillators of the substrate bath. For the sake of simplicity, we neglect the effects of spin on the properties of the SMT. The model Hamiltonian for the system is given by


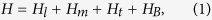


The first term *H*_*l*_ describes the Hamiltonian for the source (*l* = *S*) and the drain 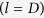 and is given by


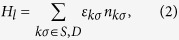


where 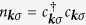 is the number operator for conduction electrons in the continuum states of the source and drain with wave vector ***k***, spin *σ*, energy *ε*_***k**σ*_ and density of states *g*_*S*(*D*)_(*ε*), 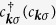 being the electron creation (annihilation) operator in the state (***k***,). The second term *H*_*m*_ describes the Hamiltonian of the molecule and is given by


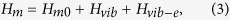


where *H*_*m*0_ is the Hamiltonian for the electronic part of the molecule and reads as


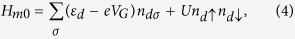


with *n*_*dσ*_ as the number operator corresponding to the electrons on the molecule with *ε*_*d*_ as the onsite energy (that can be varied experimentally by tuning the gate voltage (*V*_*G*_) and *U* is the local coulomb correlation strength. *H*_*vib*_ describes the vibrational degree of freedom of the molecule of mass *m*_0_ and frequency *ω*_0_ and can be written as,


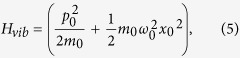


and *H*_*vib*−*e*_ represents the *el-ph* interaction on the molecule and is given by


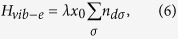


with *λ* as the *el-ph* coupling constant. The leads-molecule hybridization term with hybridization strength *V*_***k***_ is given by,


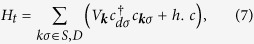


Finally, the damping effect is incorporated in (1) by introducing the term


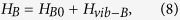



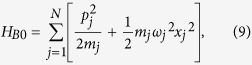



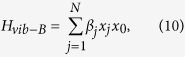


where *x*_0_ and {*x*_*j*_} are the molecular and the bath oscillator degrees of freedom and *β*_*j*_ is the coupling strength between the molecular oscillator and the *j*th bath oscillator. The oscillator bath is fully characterized by a spectral function *J*(*ω*) given by


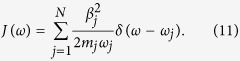


Eliminating the linear oscillator-bath interaction, we can write


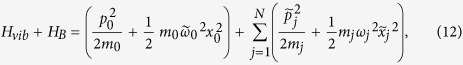


where


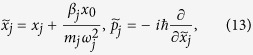



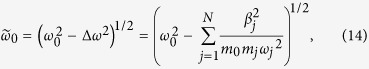


where Δ*ω*^2^ is the shift in the square of the molecular oscillator frequency caused by the linear oscillator-bath coupling. For very large *N* we can replace the summation over *j* by an integration over *ω*_*j*_. Δ*ω*^2^ can be written as


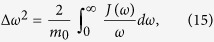


where *J*(*ω*) is the spectral function for which we choose the Lorentz-Drude form:


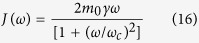


where 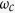 is the cutoff frequency which is much larger than the other frequencies in the system and 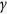 is the damping rate. The shift in the molecular frequency turns out be, 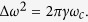 The total Hamiltonian finally reduces to


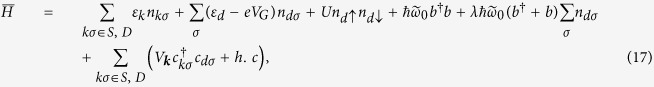


where, *b*^†^(*b*) is the creation (annihilation) operator for a molecular phonon of frequency 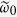. It may be noted that we have neglected the decoupled bath-oscillator Hamiltonian because that merely contributes a constant to the energy.

### Polaron Transformation

To investigate the effects of the polaronic interactions in the system, a Lang-Firsov transformation^25^ with the generator *S* = *λ*(*b*^†^ − *b*)∑_*σ*_*n*_*dσ*_, is applied to the Hamiltonian. The transformed Hamiltonian 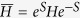 reads


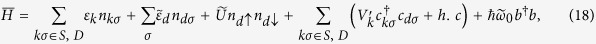


with


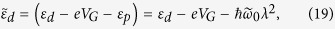



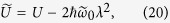



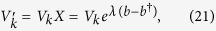


where 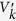 the phonon is mediated hybridization strength, 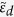 is the renormalized molecular energy level due to the polaronic effect and *ε*_*p*_ is the polaron binding energy.

### Tunneling Current

In this section, we shall employ the method of Chen *et al.*^26^. The current expression through the interacting region coupled to two metallic leads can be expressed as^27^,^28^,


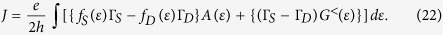


Similarly, the occupation number of the molecule are given by,


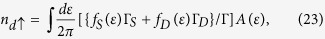


where *f*_*S*,*D*_(*ε*) are the Fermi distribution functions of the source and drain whose chemical potentials are related to the bias voltage (*V*_*B*_) and mid-voltage (*V*_*m*_) as (*μ*_*S*_ − *μ*_*D*_) = *eV*_*B*_ and (*μ*_*S*_ + *μ*_*D*_)/2 = *eV*_*m*_. For symmetric coupling,


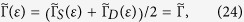


where,


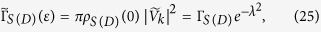


where 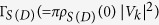 is the coupling strength between the molecule and the source (drain), *ρ*_*S*(*D*)_ being the density of states in the source (drain) channel. Here we have considered constant density of states in the source and drain. The possible excitation energy spectrum is described by the quantity called Spectral (SP) function, which is defined as


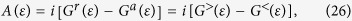


where,


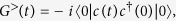



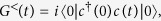



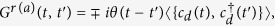


where the superscript ‘>’ (‘<’) refers to greater (lesser), ‘*r*’ (‘*a*’) refers to retarded (advanced) and 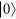 represents the true electronic ground state of the system. Where *G*^*r*(*a*)^(*ε*) and *G*^>(<)^(*ε*) are the energy-dependent retarded (advanced) and lesser (greater) electron Green’s functions of the molecule respectively. The retarded and advanced Green functions can be easily calculated using the equation of motion approach. One obtains


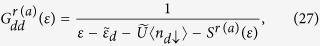


where the retarded (advanced) self-energy *S*^*r*(*a*)^(*ε*) due to hybridization interaction is given by


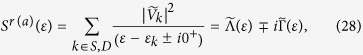


where the real part of the self-energy can be absorbed into the molecular energy level. We assume that 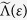 and 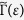 are constants within the flat band limit. The phonon operator *X* in the Hamiltonian Eq. (18) can be absorbed into a renormalized electron annihilation 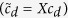 and creation 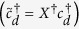 operators in the molecule region. So the Hamiltonian Eq. (18) is the usual resonant tunneling Hamiltonian with the dressed molecule electron operators. The interacting lesser Green’s function for the electrons on the molecule can be written as,


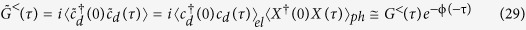



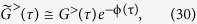


where the factors 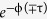 which arise from the phonon averages are given by^29^


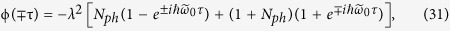



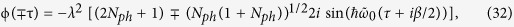


with 
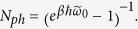
 By using the relation, 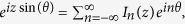 where, 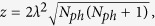 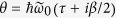.


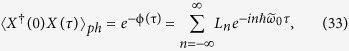


with


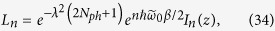


where *I*_*n*_(*z*) is the *n*th order Bessel function of complex argument. The lesser and greater Green functions can be expanded as


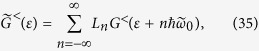



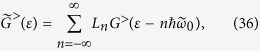


From Eq. (26) the SP function can be written as,


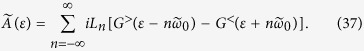


Using Keldysh formalism we can write lesser and greater Green’s functions as


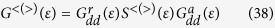


with


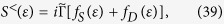



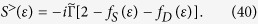


After calculating the lesser and greater Green functions we can obtain the SP function of the molecule electron using Eq. (37).

## Results and Discussion

**Figure 2:**
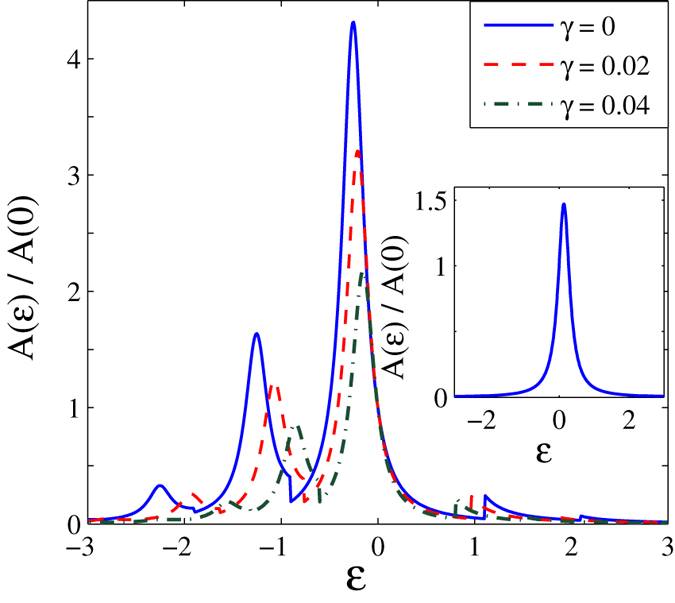
The dimensionless spectral (SP) function as a function of energy with *μ*_*S*_ = *μ*_*D*_ = 0, *λ* = 0.6. (Inset: SP function for *λ* = *γ* = 0).

In the present calculation, all energies are measured in units of phonon energy 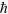
*ω*_0_ which is set equal to 1. Furthermore, the coupling of the molecule with the source and that with the drain are considered symmetric. In our calculation we have taken 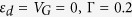 and *eV*_*m*_ = 0.1. Our main aim is to study the damping effect of the substrate on the properties of the SMT system. In Fig. 2, we show the variation of the SP function *A*(*ε*) of a SMT with *ε* for different values of the damping rate *γ* and a given value of the *el-ph* coupling constant *λ* (*λ* = 0.6). The inset shows the *A*(*ε*) vs. *ε* behavior for the case: *λ* = *γ* = 0, which is a simple Lorentzian with a single resonant peak at *ε*_*d*_ = 0. The *el-ph* interaction induces polaronic effects that renormalize the SMT parameters and shift the *ε*_*d*_ = 0 peak of the SP function towards red and also make them sharper. Most importantly, the SP function also develops side peaks at 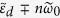 in the presence of the *el-ph* interaction. These, so called, phonon side bands in the SP function at zero temperature represent the phononic excitation energy levels created by the electrons tunneling on to the molecule by absorbing or emitting phonons. Due to the damping effect of the substrate the phonon frequency gets renormalized to 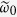. As the damping rate increases, the heights of the phonon side bands decrease and broaden. This suggests that as the damping rate increases, the occupation probability of the phonon side bands decreases.

**Figure 3:**
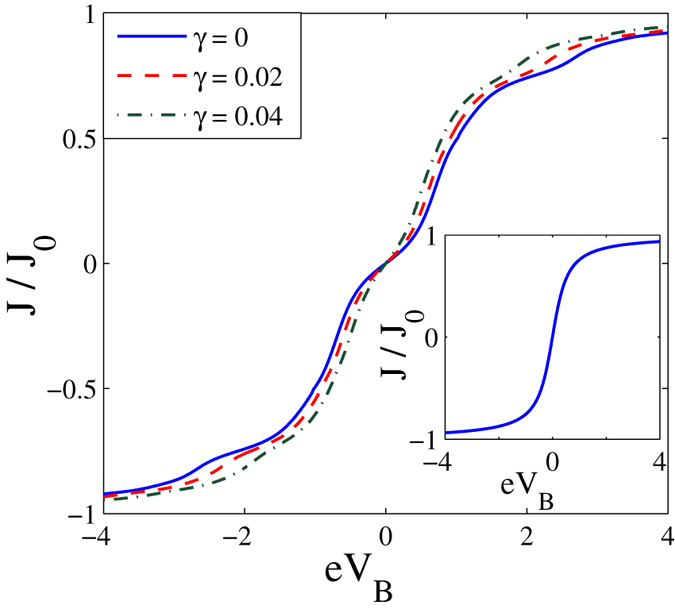
The dimensionless current *J*/*J*_0_ as a function of bias voltage *eV*_*B*_ with *λ* = 0.6. (Inset: Current for *λ* = *γ* = 0).

**Figure 4:**
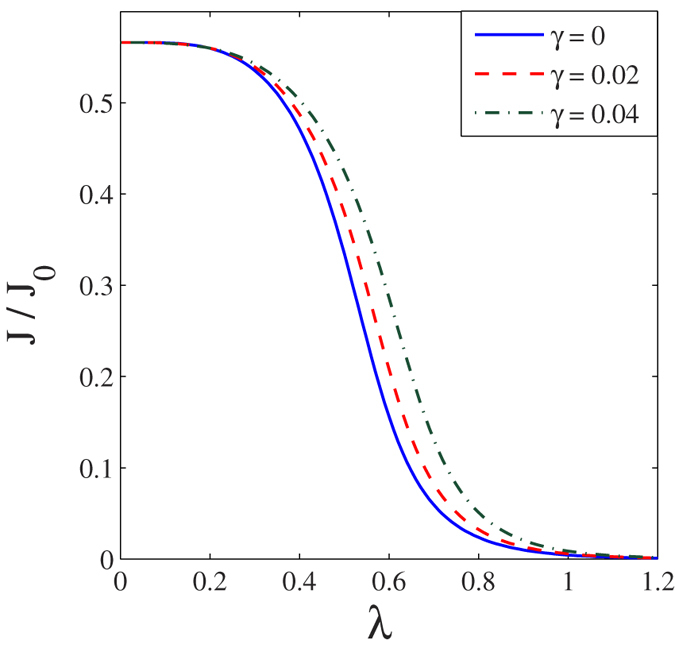
The dimensionless current as a function of *λ* with *eV*_*B*_ = 0.5.

In Fig. 3 we present the results for the normalized tunneling current *J*(*eV*_*B*_, *eV*_*m*_) of the SMT system as a function of bias voltage for different values of *γ* in the presence of the *el-ph* interaction. The normalized tunneling current for the case: *λ* = *γ* = 0 is shown in the inset for comparison. As the damping rate increases the current also increases. To see the effect of *el-ph* interaction on current, we plot, in Fig. 4, the current as a function of *λ* for different values of *γ* at constant bias voltage *V*_*B*_. As *λ* increases, the current decreases smoothly but rapidly and becomes zero at a critical value of *λ*, say *λ*_*c*_. This is easy to understand physically, though mathematically, Eq. (25) explicitly shows that the current decreases exponentially with *λ*. At the same time as *λ* increases, the separation between the molecule energy level and the chemical potential of source increases. As *γ* increases, *λ*_*c*_ is also found to increase.

**Figure 5:**
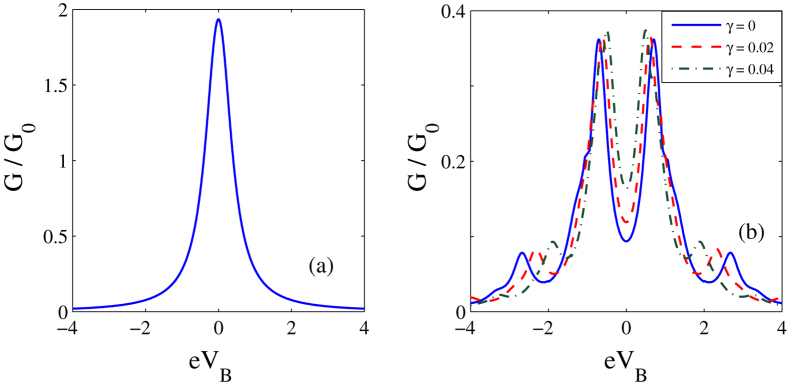
Differential conductance as a function of Bias voltage. (**a**) 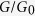 vs *eV*_*B*_ for *γ* = *λ* = 0. (**b**) *G*/*G*_0_ vs *eV*_*B*_ for *λ* = 0.6 and 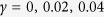.

**Figure 6:**
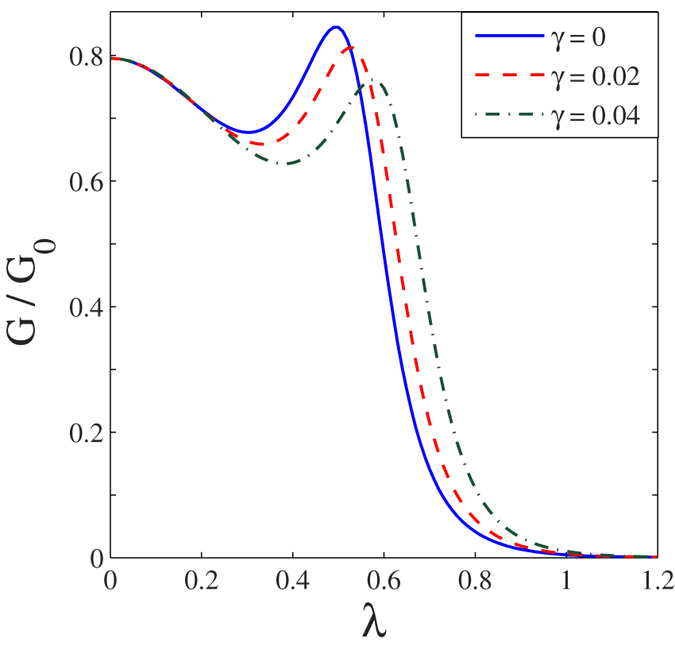
*G*/*G*_0_ as a function of *λ* for a few values of *γ* with *eVB* = 0.6.

In Fig. 5, we present the results for the differential conductance *G*(*eV*_*B*_, *eV*_*m*_)(=*dJ*/*dV*_*B*_) as a function of the bias voltage. In Fig. 5(a) we show the behaviour of *G* in the absence of the *el-ph* interaction and the damping effect. The symmetry in the conductivity peaks is clearly visible. In Fig. 5(b) we show the behavior of *G* in the presence of *el-ph* interaction (*λ* = 0.6) for different values of *γ*. The *el-ph* interaction and damping have visible independent effects on *G*. The effect of the *el-ph* interaction is two-fold. First, it sharpens the conductivity peaks and secondly and more importantly it gives rise to new satellite peaks that originate because of phonon-assisted tunneling transport. To show the effect of *el-ph* interaction on the differential conductance explicitly, we plot in Fig. 6, *G*/*G*_0_ as a function of *λ* for different values of *γ*. For a given *γ*, the differential conductance has a peak at a certain value of *λ* and as *γ* increases the peak reduces in height and also shifts towards right. Both shifting and reduction in height of the conductance peaks are the expected behavior.

**Figure 7:**
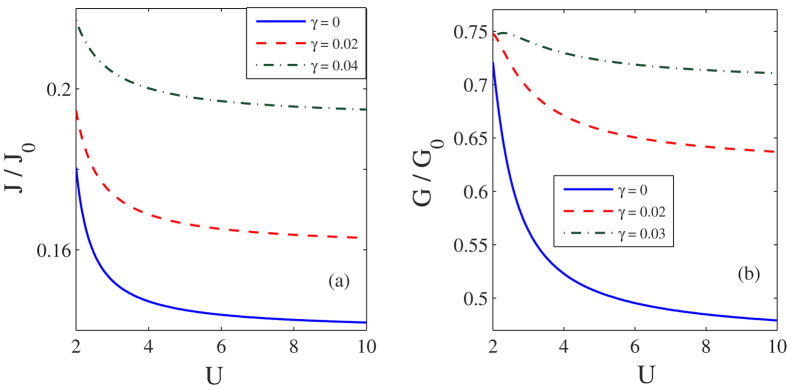
Effect of *el-el* interaction strength on current and differential conductance for *λ* = 0.6. (**a**) *J*/*J*_0_ vs *U* for 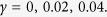 (**b**) *G*/*G*_0_ vs *U* for *λ* = 0, 0.02, 0.03.

In Fig. 7(a,b) we plot *J* and *G* respectively as a function of the *el-el* interaction strength *U*. As *U* increases, both *J* and *G* decrease, while they increase with increasing *γ*. These variations can be explained in the following way. Due to the Coulomb blockade effect the onsite coulomb correlation opposes the double occupancy on the molecule as a result of which the current decreases with increasing *U*, while as *γ* increases, the effective phonon frequency decreases leading to an increase in the current and the conductance.

**Figure 8:**
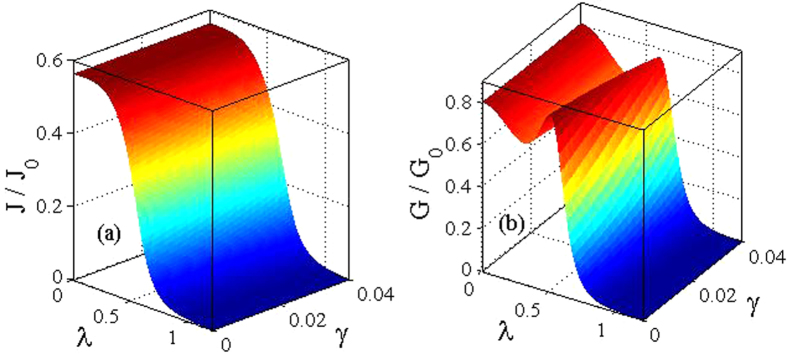
Three dimensional plots of current and differential conductance for *eV*_*B*_ = 0.5. (**a**) *J*/*J*_0_ as a function of both *λ* and *γ*. (**b**) *G*/*G*_0_ as a function of both *λ* and *γ*.

Finally, we make a three-dimensional plot for *J* as a function of both *γ* and *λ* in Fig. 8(a) and for *G* as a function of *γ* and *λ* in Fig. 8(b).

## Conclusion

In this work we have considered a SMT system in which a molecule or a quantum dot is placed on a substrate coupled to two metal leads acting as a source and a drain. The system is modeled by the AH Hamiltonian with a linear Caldeira-Leggett term to include the linear coupling between the substrate and the molecule which describes the damping effect. We have calculated the spectral function, tunneling current and differential conductance of the SMT system using the Keldysh Green function method. We have also analyzed the effect of *el-ph* interaction and the damping effect due to the substrate. We have shown that the *el-ph* interaction induces polaronic effects that renormalize the SMT parameters and shift the peak of the spectral function towards red and also make them sharper. The spectral function also develops side bands whose heights decrease and the widths broaden with increasing damping rate. As *λ* increases, the tunneling current is found to decrease smoothly but rapidly to zero at a critical value of *λ*. The *el-ph* interaction also sharpens the conductivity peaks and gives rise to new satellite peaks that originate because of phonon-assisted tunneling transport. It is also shown that the local *el-el* interaction causes a reduction in both the tunneling current and the differential conductance. Due to the damping effect of the substrate, the effective phonon frequency of the molecule oscillator decreases as a result of which the tunneling current increases. In the presence of *el-ph* and *el-el* interactions and damping, the differential conductance exhibits an interesting behavior. The spin-orbit interaction may also have an interesting effect on the tunneling current and the differential conductance. This issue is presently under investigation and the results will be published in due course. Which will be the subject matter of a future investigated.

## Additional Information

**How to cite this article**: Raju, C. N. and Chatterjee, A. Quantum dissipative effects on non-equilibrium transport through a single-molecular transistor: The Anderson-Holstein-Caldeira-Leggett model. *Sci. Rep.* **6**, 18511; doi: 10.1038/srep18511 (2016).
